# *BMC Structural Biology* reviewer acknowledgment 2014

**DOI:** 10.1186/s12900-015-0029-1

**Published:** 2015-02-17

**Authors:** Catherine J Potenski

**Affiliations:** BioMed Central, 233 Spring Street, New York, NY 10013-1578 USA

**Oluwatoyin Asojo**

USA

**Shannon Au**

Hong Kong

**Lin Bai**

USA

**Douglas Barrick**

USA

**Nir Ben-Tal**

Israel

**Andrea Bernini**

Italy

**David Bevan**

USA

**Martino Bolognesi**

Italy

**Mohammad Reza Bozorgmehr**

Iran

**David Burke**

UK

**Teresa Carlomagno**

Germany

**Rita Casadio**

Italy

**Sudha Chakrapani**

USA

**Chi-Cheng Chiu**

Taiwan

**Patricia Coltri**

Brazil

**Lisa Craig**

Canada

**Nora Cronin**

UK

**Joel Eissenberg**

USA

**Jianwen Fang**

USA

**Diego Ferreiro**

Argentina

**Slawomir Filipek**

Poland

**Federico Fogolari**

Italy

**Dmitrij Frishman**

Germany

**Qingshan (Bill) Fu**

USA

**Bruce Gelb**

USA

**Vince Grolmusz**

Kuwait

**Nam-Chul Ha**

Korea, South

**Ulrich Hansmann**

USA

**Bart Hazes**

Canada

**Ronald Hills**

USA

**Gunnar Jeschke**

Switzerland

**Rameshwar Kadam**

USA

**Brian Kelch**

USA

**Chu-Young Kim**

Singapore

**Seiichiro Kishishita**

Japan

**Stephan König**

Germany

**Andriy Kryshtafovych**

USA

**Roman Laskowski**

UK

**Vanessa Leone**

USA

**Elena Levin**

USA

**Zhijun Li**

USA

**Christian Linke-Winnebeck**

Germany

**George Lountos**

USA

**Jianzhu Ma**

USA

**Francisco Martinez**

Spain

**Yves Mechulam**

France

**Daniela Monti**

Italy

**Giulia Morra**

Italy

**Marvin Nieman**

USA

**Poul Nissen**

Denmark

**Diederik Opperman**

South Africa

**Yingjie Peng**

USA

**Robert Rambo**

USA

**Daniel Roche**

France

**Seong Eon Ryu**

Korea, South

**Wolf-Dieter Schubert**

South Africa

**Blake Simmons**

USA

**Heinz-Juergen Steinhoff**

Germany

**Eric Sundberg**

USA

**Dazhi Tan**

USA

**Silvio Tosatto**

Italy

**Tai Wang**

Switzerland

**Sheng Ye**

China

**Yaroslava Yingling**

USA

